# Human Blood Concentrations of Cotinine, a Biomonitoring Marker for Tobacco Smoke, Extrapolated from Nicotine Metabolism in Rats and Humans and Physiologically Based Pharmacokinetic Modeling

**DOI:** 10.3390/ijerph7093406

**Published:** 2010-09-01

**Authors:** Hiroshi Yamazaki, Kana Horiuchi, Ryohji Takano, Taku Nagano, Makiko Shimizu, Masato Kitajima, Norie Murayama, Fumiaki Shono

**Affiliations:** 1 Laboratory of Drug Metabolism and Pharmacokinetics, Showa Pharmaceutical University, 3-3165 Higashi-Tamagawa Gakuen, Machida, Tokyo 194-8543, Japan; E-Mails: kana.horiuchi@shionogi.co.jp (K.H.); takano.r@jp.fujitsu.com (R.T.); doutai@ac.shoyaku.ac.jp (T.N.); shimizu@ac.shoyaku.ac.jp (M.S.); muraya_n@ac.shoyaku.ac.jp (N.M.); 2 High Technology Research Center, Showa Pharmaceutical University, 3-3165 Higashi-Tamagawa Gakuen, Machida, Tokyo 194-8543, Japan; 3 Fujitsu Kyusyu Systems, 2-2-1 Momochihama, Sawara-Ku, Fukuoka 814-8589, Japan; E-Mail: kitajima.masato@jp.fujitsu.com (M.K.); 4 Japan Chemical Industry Associations (JCIA), 1-4-1 Shinkawa, Chuo-Ku, Tokyo 104-0033, Japan; E-Mail: fshono@jcia-net.or.jp

**Keywords:** physiologically based biokinetic modeling, cytochrome P450, simulation, no-observed-adverse-effect level, biomonitoring, human liver microsomes

## Abstract

The present study defined a simplified physiologically based pharmacokinetic (PBPK) model for nicotine and its primary metabolite cotinine in humans, based on metabolic parameters determined *in vitro* using relevant liver microsomes, coefficients derived *in silico*, physiological parameters derived from the literature, and an established rat PBPK model. The model consists of an absorption compartment, a metabolizing compartment, and a central compartment for nicotine and three equivalent compartments for cotinine. Evaluation of a rat model was performed by making comparisons with predicted concentrations in blood and *in vivo* experimental pharmacokinetic values obtained from rats after oral treatment with nicotine (1.0 mg/kg, a no-observed-adverseeffect level) for 14 days. Elimination rates of nicotine *in vitro* were established from data from rat liver microsomes and from human pooled liver microsomes. Human biomonitoring data (17 ng nicotine and 150 ng cotinine per mL plasma 1 h after smoking) from pooled five male Japanese smokers (daily intake of 43 mg nicotine by smoking) revealed that these blood concentrations could be calculated using a human PBPK model. These results indicate that a simplified PBPK model for nicotine/cotinine is useful for a forward dosimetry approach in humans and for estimating blood concentrations of other related compounds resulting from exposure to low chemical doses.

## 1. Introduction

It has been argued internationally that appropriate use of human biomonitoring information should be made in risk assessments when creating public policy [1,2] Development and dissemination of chemical-specific methods and basic information is necessary to interpret biomonitoring results and to promote risk-based decision making [3,4]. It is of global interest to develop more advanced and accurate risk assessment systems to support appropriate interpretation and communication based on human biomonitoring results [5]. Pharmacokinetic and/or toxicokinetic parameters for a variety of chemicals have been determined in animal toxicology studies, even when limited corresponding data exist for humans [6]. Species differences of drug-metabolizing enzymes in the liver, including cytochrome P450 enzymes, are the focus for understanding qualitative and quantitative differences in blood concentrations or chemical exposures in animals and in humans [7]. It has been generally attempted to collect extensive information regarding specific physiologically based pharmacokinetic (PBPK) models found in the literature for predicting concentrations in various biological fluids following multiple dose exposures [4]. However, although simple, inexpensive, and reliable methods are needed for evaluating the accurate toxic risk, as only very complicated models have been established so far [8].

Conventional smoking and environmental tobacco smoke have significant health effects [9–12]. Levels of cotinine, a metabolite of nicotine [13–15], in the blood track exposure to tobacco smoke [9]. In the past 15 years, it has been reported that blood cotinine levels for nonsmokers in the United States population have decreased by about 70%, indicating that public health interventions to reduce exposure have been successful (http://www.cdc.gov/exposurereport/), but such information for other groups or countries is limited [16,17]. Recently we reported that the biomonitoring of cotinine in urine was a good, easy-to-use marker for plasma levels of the sum of nicotine metabolites in Japanese smokers, independent of genetic polymorphism of the nicotine-metabolizing enzyme, P450 2A6 [18].

**Figure 1.**
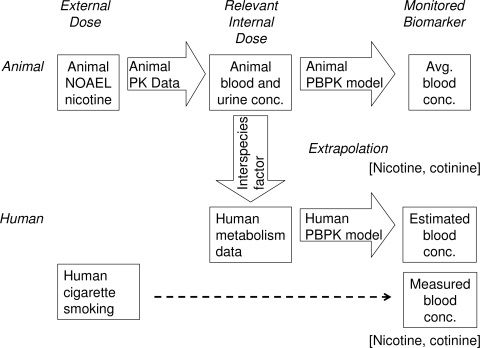
Approach for calculating blood-based biomonitoring equivalents for nicotine. PK, pharmacokinetics. Biomonitoring of nicotine and cotinine in plasma was carried out in male Japanese smokers.

Therefore, the purpose of the present study was to carry out a forward dosimetry approach (shown in Figure 1), using data from chemical doses administered to animals to predict their concentrations in humans. As test substances, nicotine and its primary metabolite cotinine were selected because as mentioned, they are widely used as biomarkers for tobacco smoke, in spite of the complex metabolic fate of nicotine [19,20]. We report herein that the adjusted animal biomonitoring equivalents after orally administered doses at a no-observed-adverse-effect level (NOAEL) in rat studies were scaled to human biomonitoring equivalents using known species allometric scaling factors and human metabolic data with a simple PBPK model. A typical study of the biomonitoring of nicotine and cotinine in the a representative pooled blood of Japanese smokers [18] supported the PBPK model used in the present study.

## 2. Experimental Section

### 2.1. Chemicals, Animals, and Enzyme Preparations

Nicotine and cotinine were obtained from Wako Pure Chemicals (Osaka, Japan). Male rats (7 weeks old) were treated daily with nicotine (1.0 mg/kg body weight) orally for 14 days or interperitoneally for 3 days, based on a NOAEL dose [21]. This study was approved by the experimental animal committee of Showa Pharmaceutical University. Liver microsomes from male Sprague-Dawley rats (7 weeks old) treated with nicotine (1.0 mg/kg) and from untreated controls were prepared as described previously [22]. Microsomal P450 contents were determined spectrally by the established method [23,24]. Protein concentrations were measured by using a bicinchoninic acid (BCA) protein assay kit (Pierce, Rockford, IL, USA). Pooled liver microsomes from humans were obtained from BD Biosciences (Woburn, MA, USA). Typical P450 substrates, their reaction products, and other reagents used in this study were obtained from the sources described previously or were of the highest quality commercially available [22,25].

Typical P450-dependent marker oxidation activities were measured in liver microsomes in rats to evaluate enzyme inductions on treatment with nicotine. Activities for the *O*-dealkylation of ethoxyresorufin (20 μM, for P450 1A) and pentoxyresorufin (100 μM, P450 2B) and for testosterone 7α-hydroxylation (200 μM, P450 2A), tolbutamide methyl hydroxylation (1,000 μM, P450 2C), bufuralol 1′-hydroxylation (20 μM, P450 2D), chlorzoxazone 6-hydroxylation (50 μM, P450 2E), and midazolam 1′- and 4-hydroxylation (100 μM, P450 3A) were assayed according to the described high performance liquid chromatography methods [22,26,27].

### 2.2. Nicotine and Cotinine Determinations in Biological Samples from Rats and Human Smokers

This study was approved by the ethics committee of Showa Pharmaceutical University. Blood and urine samples from individual rats and pooled human plasma from five male Japanese smokers (22–44 years old [18]) were diluted 10-fold with water. Nicotine and cotinine concentrations in these samples were measured by a liquid chromatography/mass spectrometry (LC/MS) system [28]. A Quattro micro API mass analyzer (Waters, Tokyo, Japan) was operated in the electrospray positive ionization mode and was directly coupled to a Waters LC 2695 system with an octadecylislane C_18_ column (Atlantis, 3 μm, 2.1 mm × 100 mm) and MassLynx NT4.1 software for data acquisition (Waters). To tune the mass spectrometer, the cone voltage was optimized to maximize the intensity of the precursor ions for nicotine (*m/z =* 163) and cotinine (*m/z* = 177). The collision energy was then adjusted to optimize the signal. Typical tuning conditions were as follow: electrospray capillary voltage, 3.2 kV; sample cone voltage for nicotine and cotinine, 28 and 38 V; and collision energy, 19 and 23 eV, respectively, at a collision gas (Ar) pressure of 1.6 × 10^−4^ kPa. The gradient mobile phase consisted of 50 mM ammonium formate (pH 5.0) and CH_3_CN (v/v): 0–3 min with 5% CH_3_CN (v/v) in 50 mM ammonium formate; for 3–8 min with 5%–30% CH_3_CN (v/v); for 8–11 min with 30% CH_3_CN; and for 11–18 min with 30%–5% CH_3_CN (v/v), at a flow rate of 0.25 mL min^−1^.

### 2.3. Human Metabolic Study

Elimination rates of nicotine in liver microsomes from humans and rats in the presence of cytosolic fractions were measured by the LC/MS system mentioned above and were compared. Briefly, a typical incubation mixture consisted of 100 mM potassium phosphate buffer (pH 7.4), an NADPH-generating system, a substrate (1.0 μM), and liver microsomes (0.50 mg protein/mL) and cytosol (0.70 mg protein/mL, to yield cotinine, [29]) in a final volume of 0.25 mL. Incubations were carried out at 37 ºC for 30 min. The assay linearity with respect to time and protein concentration and the reproducibility (within <15%) were confirmed. The incubation was terminated by adding 0.40 mL of ice-cold acetonitrile.

### 2.4. Estimation of Nicotine/Cotinine Concentrations by PBPK Modeling with Suitable Parameters

**Figure 2.**
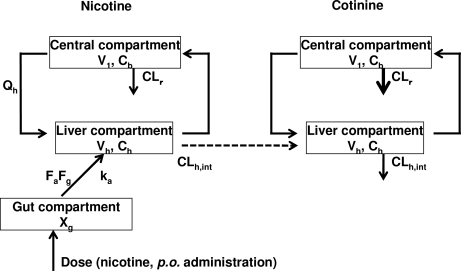
PBPK model established in this study for rats and humans.

A simplified PBPK model was set up as described previously [30,31]. Parameter values for the physicochemical properties of compounds (f_u,p_, logP, K_p,h_, and R_b_) are shown in Table 1. Values of f_u,p_ and logP were obtained by *in silico* estimation using SimCYP and ChemDrawBioUltra software [32]; K_p,h_ was estimated from these two values (Appendix A), and R_b_ was assumed to be 1.0 (blood and plasma concentrations are assumed to be equal). Parameter values which represent the physiological properties such as hepatic volumes and blood flow rate in rats or humans were taken from the literature [30]. Experimental plasma concentrations of compounds were analyzed by WinNonlin software (Professional version 5.01) with a one-compartment model and yielded primary k_a_ and k_el_ values as pharmacokinetic parameters (abbreviations used are also shown in Table 1). Values of total clearance (CL_tot_), hepatic clearance (CL_h_), CL_h,int_, and V_1_ were also calculated from the results of one-compartmental model (Appendix B). Subsequently, final parameter values (k_a_, CL_h,int_ and V_1_) for the rat PBPK model were calculated using the initial values mentioned above by the user model in WinNonlin and are shown in Table 1. Consequently, the following systems of differential equations were solved to conduct the concentrations in each compartment shown in Figure 2.

For nicotine:

$$\begin{array}{c} \frac{{dX}_{g}(t)}{dt}=-k_{a}\cdot X_{g}(t)\;\;\;\text{where}\;\text{at}\;t=0,\;X_{g}(0)=\text{Dose}\\ V_{h}\frac{{dC}_{h}}{dt}={\text{Q}}_{h}\cdot C_{b}-\frac{{\text{Q}}_{h}\cdot C_{h}\cdot R_{b}}{K_{p,h}}+k_{a}\cdot X_{g}-{CL}_{h,\text{int}}\cdot \frac{C_{h}}{K_{p,h}}\cdot f_{u,p}\\ V_{1}\frac{{dC}_{b}}{dt}=-{\text{Q}}_{h}\cdot C_{b}+\frac{{\text{Q}}_{h}\cdot C_{h}\cdot R_{b}}{K_{p,h}}-{CL}_{r}\cdot C_{b} \end{array}$$

where *X_g_* is the substance amount in gut, *C_h_* is the hepatic substance concentration, and *C_b_* is the blood substance concentration.

For cotinine:

$$\begin{array}{c} V_{1}\frac{{dC}_{b}}{dt}=-{\text{Q}}_{h}\cdot C_{b}+\frac{{\text{Q}}_{h}\cdot C_{h}\cdot R_{b}}{K_{p,h}}-{CL}_{r}\cdot C_{b}\\ V_{h}=\frac{{dC}_{h}}{dt}={\text{Q}}_{h}\cdot C_{b}-\frac{{\text{Q}}_{h}\cdot C_{h}\cdot R_{b}}{K_{p,h}}-{CL}_{h,\text{int}}\cdot \frac{C_{h}}{K_{p,h}}\cdot f_{u,p}+{CL}_{h,\text{int},\text{ni}\text{cot}\text{ine}}\cdot \frac{C_{h,\text{ni}\text{cot}\text{ine}}}{K_{p,h,\text{ni}\text{cot}\text{ine}}}\cdot f_{u,p,\text{ni}\text{cot}\text{ine}} \end{array}$$

To define a simplified PBPK model for nicotine and cotinine in humans based on the rat PBPK model, we used relevant liver microsomes and physiological parameters (CL_r_, k_a_, and V_1_) and applied the systems approach to fit them into the traditional parallelogram (animal scale up strategy) for risk assessment [4], as shown in Figure 1 (Appendix C). The *in vivo* hepatic intrinsic clearance (CL_h,int_) of nicotine in humans was estimated by multiplying the calculated initial parameters for *in vitro* hepatic intrinsic clearance values in humans by the ratio of *in vivo* to *in vitro* hepatic intrinsic clearance in rats, as mentioned above for modeling in rats. Then, the final parameters for PBPK modeling in humans were calculated are shown in Table 2. As was done for the rat model, systems of differential equations were solved to achieve concentrations in each compartment in humans.

## 3. Results and Discussion

**Figure 3.**
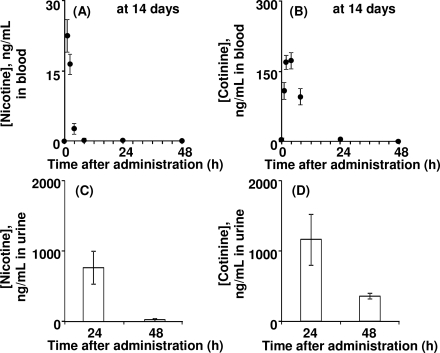
PK profiles in rats treated with nicotine. Nicotine (A, C) and cotinine (B, D) concentrations in blood (A, B) and urine (C, D) were determined in rats treated with nicotine (1 mg/kg/day) after the final administration of 14 daily doses.

To obtain detailed PBPK model parameters, male rats were orally treated with nicotine according to the protocol for general repeated exposure tests. Figure 3 shows the mean levels of nicotine and cotinine in blood and urine from rats after the final treatment of 14 daily repeated doses of nicotine (1.0 mg/kg). Nicotine was rapidly absorbed and immediately cleared within a half day (Figure 3A). Nicotine was extensively biotransformed to cotinine; cotinine elimination from the blood seemed to be slower than that of nicotine (Figure 3B). Urinary excretion of nicotine and cotinine was almost complete within 24 h after the final repeated administration (Figures 3C, 3D). Renal clearance (CL_r_) values of nicotine and cotinine were calculated from the amounts excreted into the urine (5.32 and 8.23 μg) divided by the area under the blood curves (56.5 and 1,970 μg·h/L), giving 0.0994 and 0.00421 L/h, respectively. Primary hepatic clearance values of nicotine and cotinine were obtained by subtraction of CL_r_ from total clearance. Values of the plasma unbound fraction (f_u,p_) of nicotine and cotinine were calculated to be 0.688 and 0.743, respectively, by *in silico* estimation with SimCYP (Table 1).

**Figure 4.**
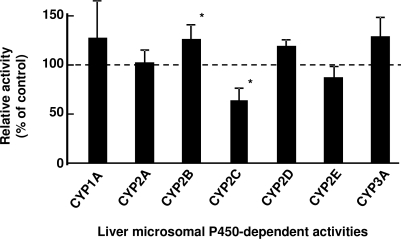
Liver microsomal P450-dependent activities after nicotine treatment. Control activities were taken from liver microsomes from untreated rats. Data columns with bars present means ± SDs (n = 4). Significant differences compared with the control activities: *p < 0.05.

**Figure 5.**
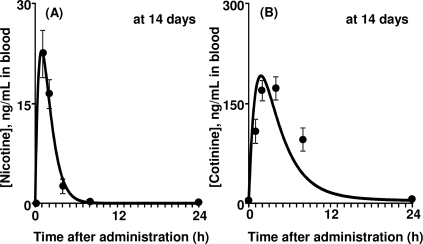
Measured and estimated blood concentrations in rats after oral administration of nicotine (A) and cotinine (B) for 14 days. Data points with bars represent means ± SDs (n = 5). The curves show the concentrations estimated by PBPK modeling.

P450 induction in rat liver microsomes was investigated after intraperitoneal treatment with nicotine for 3 days (Figure 4). Judging from the typical P450-dependent drug oxidation activities, CYP2B- and CYP2C-mediated activities were slightly increased and decreased, respectively (Figure 4) as similarly described [33,34], suggesting that P450 induction or suppression by repeated treatments with nicotine was almost negligible in nicotine metabolism mediated mainly by several common and uncommon P450 isoforms in rats and humans. Consequently, final parameters such as hepatic intrinsic clearance (CL_h,int_), volume of systemic circulation (V_1_), and absorption rate constant (k_a_) for the rat PBPK model were recalculated from the primary values by the user-model in WinNonlin to give 5.44 L/h, 0.746 L, and 1.07 h^−1^ and are shown in Table 1. By running the rat PBPK model system shown in Figure 2, the blood concentration curves of nicotine and cotinine were estimated after repeated oral administration with 0.25 mg of nicotine to a rat (250 g bw); the curves are shown in Figure 5. These estimated *in silico* concentration curves of nicotine and cotinine are shown with the experimental *in vivo* data points.

It has been reported that the ratios of *in vitro* over *in vivo* intrinsic clearance values have little species differences [35]. Hepatic clearance of nicotine *in vitro* was determined in pooled human liver microsomes and compared with data from liver microsomes from rats pretreated with nicotine and from untreated controls (Table 3). Hepatic clearance of nicotine in human liver microsomes was calculated to be 6.7 μL/min/mg protein; this was similar to the values obtained for rat livers. Subsequently, hepatic intrinsic clearance of nicotine was found to be 24 L/h in an *in vitro* study using the biological coefficients already established. The intrinsic clearance values of nicotine based on rat *in vivo* (Table 1) and rat *in vitro* (Table 3) experiments were different; this ratio (5.44/0.173) was used as the compensating factor for estimating *in vivo* hepatic intrinsic clearance in humans. Finally, a value of 755 L/h for the nicotine hepatic intrinsic clearance (CL_h,int_) was adopted to represent the *in vivo* status in the final human PBPK model, the parameters of which are shown in Table 2. It should be mentioned that extensive hepatic clearance of nicotine assumed to be almost equal to and dependent on hepatic blood flow rate.

**Figure 6.**
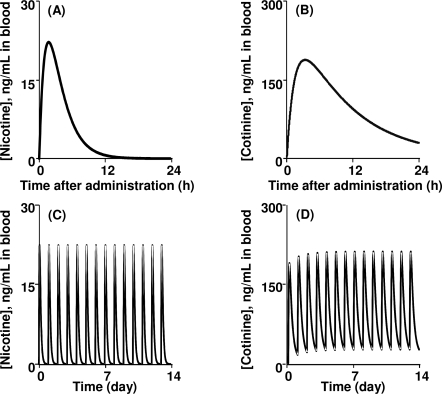
Nicotine (A, C) and cotinine (B, D) concentrations modeled in humans after single (A, B) or multiple (C, D) oral administration of nicotine (1 mg/kg/day) estimated using the PBPK model. Only limited accumulation was observed for multiple doses.

Figure 6 indicates the estimated human blood concentrations of nicotine and cotinine after modeling single and repeated oral administration with nicotine (1.0 mg/kg). The apparent maximum concentrations of nicotine and cotinine were estimated to be approximately 20 and 180 ng/mL, respectively. Our estimated CL_tot_ values for nicotine in rats and humans were calculated to be 2.8 L/h/kg in rats and 81.5 L/h in humans, respectively, which were consistent with the reported CL_tot_ values of 3.0 L/h/mg [36] in rats and 79.1 L/h [37] in humans. When daily administration of nicotine was modeled for 14 days, some accumulation of cotinine (approximately 20 ng/mL) was estimated by the present human PBPK model.

Five male Japanese smokers provided information on the numbers of cigarettes smoked daily (mean, 31.0) [8]; using a value of 1.4 mg nicotine intake per cigarette [38], the estimated daily nicotine intake was 43.4 mg. This was one of the repetitive pooled samples for biomonitoring of plasma levels of nicotine and cotinine conducted after daily cigarette smoking in a population of 92 male Japanese smokers with a mean age of 37 years who had smoked an average of 23 cigarettes per day for 16 years [8]. Nicotine and cotinine concentrations in the pooled plasma samples taken from five smokers 1 h after smoking were 15.6 and 110 ng/mL, respectively. When the intake of 43.4 mg nicotine (as one dose) through the absorption compartment was modeled in a person with a body weight of 70 kg, the estimated concentrations of nicotine and cotinine in the central compartment after 1 h from the human PBPK model were 17 and 150 ng/mL, respectively, which fell within the ranges shown in Figure 6.

It is generally accepted that PBPK modeling could be of use for understanding the relationship between chemical exposure and concentrations in body fluids (Figure 1). However, the multiple compartments and many complicated equations found in traditional PBPK modeling cause severe difficulties when applying the model. Simple and reliable methods have not yet been established, but such models are needed to explore the biological significance of a wide range of chemicals. The present study defined a simplified PBPK model for nicotine and cotinine in humans (Figure 2); the model was based on physiological parameters derived from the literature, coefficients derived *in silico*, metabolic parameters determined *in vitro* using relevant liver microsomes, and *in vivo* experiment-supported PBPK modeling in rats (Figure 5). The developed PBPK model for nicotine and cotinine in rats simply consisted of three compartments, including the gut as a chemical receptor compartment the liver as a metabolizing compartment, and the general circulation as central compartment for nicotine, and three equivalent compartments for cotinine (Figure 2). In the present model (Figure 2), chemicals are received first at the receptor compartment with the net absorption factor as 1 (namely, F_a_F_g_ = 1 indicating no first pass effects). Although the apparent gap between the oral and pulmonary uptake models might be problematic, chemical concentrations rapid absorbed from inhalation route [39] could be treated in the similar PBPK modeling system. Furthermore, oral administration of chemicals is a key route of exposures based on the toxicology testing.

Human biomonitoring is important for many aspects of environmental health [1,2]. Recently, the Centers for Disease Control and Prevention in the United State reported several pieces of relevant data, including the 95th percentile values of serum cotinine levels (∼2 ng/mL, http://www.cdc.gov/exposurereport/). Human biomonitoring data presented in the current study using pooled plasma from male Japanese smokers revealed that plasma concentrations of nicotine and cotinine could be calculated using the developed PBPK model in humans (Figure 6). In our preliminary study, cotinine was detected in some plasma samples obtained from several male Japanese nonsmokers, resulting in approximately one cigarette equivalent per day among a one-third nonsmokers. Similarly, based on our forward dosimetry approach system, in the United States, nonsmokers (including children) receive the equivalent in nicotine of less than one cigarette per day as a result of exposure to second-hand smoke.

Evaluation of the developed rat model was performed by comparing the blood concentrations predicted by PBPK modeling *in silico* and experimental pharmacokinetic values from plasma and urine obtained from rats *in vivo* after repeated oral treatment with nicotine at a no-observed-adverse-effect level. In the present study, nicotine metabolism and disposition in rats was similar to reported findings [36] by single intra-arterial treatment. To overcome the species differences in animals and humans, the traditional parallelogram technique used in systems biology [3,4] was adapted for this study to estimate the value of *in vivo* human hepatic clearance from *in vitro* data (Table 3).

## 4. Conclusions

The simplified PBPK model of nicotine and cotinine, especially in the context of biomonitoring for nicotine exposure, was developed and validated with a combination of algorithms, *in vitro* and *in vivo* experimentation and literature resources. In summary, the present study indicates that simplified PBPK modeling for nicotine and cotinine is useful for a forward dosimetry approach in rats and humans to estimate blood concentrations of nicotine and other related compounds from low chemical doses such as those at the no-observed-adverse-effect level.

## Figures and Tables

**Table 1.** Parameters used for the rat PBPK model.

**Parameter**	**Symbol**	**Nicotine**	**Cotinine**	**Unit**
Octanol-water partition coefficient	logP	0.930	0.040	
Hepatic intrinsic clearance	CL_h,int_	5.44	0.208	L/h
Liver-plasma concentration ratio	K_p,h_	0.797	0.680	-
Renal clearance	CL_r_	0.0994	0.00421	L/h
Plasma unbound fraction	f_u,p_	0.688	0.743	-
Ratio of the blood to plasma concentration	R_b_	1.00	1.00	-
Volume of systemic circulation	V_1_	0.746	0.451	L
Hepatic volume	V_h_	0.00850	0.00850	L
Hepatic blood flow rate of systemic circulation to the tissue compartment	Q_h_	0.853	0.853	L/h
Absorption rate constant	k_a_	1.07	-	h^−1^
Fraction absorbed × intestinal availability	F_a_F_g_	1.00	-	-
Dose	Dose	0.25	-	mg

**Table 2.** Parameters used for the human PBPK model.

**Parameter**	**Symbol**	**Nicotine**	**Cotinine**	**Unit**
Hepatic intrinsic clearance	CL_h,int_	755	20.6	L/h
Renal clearance	CL_r_	4.25	0.180	L/h
Volume of systemic circulation	V_1_	209	127	L
Hepatic volume	V_h_	1.50	1.50	L
Hepatic blood flow rate systemic circulation to the tissue compartment	Q_h_	96.6	96.6	L/h
Absorption rate constant	k_a_	0.795	-	h^−1^
Dose	Dose	70	-	mg

Other parameters are the same as those shown in Table 1 for the rat PBPK model.

**Table 3.** *In vitro* hepatic intrinsic clearance of nicotine determined using liver microsomes.

**Enzyme source**	**Clearance, μL/min/mg protein**	**L/h a**
Rat livers, untreated a	7.9 ± 1.4	0.142
Rat livers, treated with nicotine b	9.6 ± 1.9	0.173
Pooled human livers	6.7	24.0

Nicotine (1.0 μM) was incubated with rat or human liver microsomes in the presence of an NADPH-generating system. The reduction rates of nicotine were determined by LC/MS.

a Estimated clearance values were extrapolated using the following values: 40 mg liver microsomal protein per g liver, 10 g liver weight per 0.25 kg of rat body weight, and 1.5 kg liver per 70 kg of human body weight.

b Mean ± SD (n =4) values using liver microsomes from individual rats pretreated with nicotine (1.0 mg/kg) daily for 3 days or from untreated controls.

## Appendix A

The liver-plasma concentration ratio (*K*_p,H_) was calculated from Equation A1 [40]:

(A1) $$K_{p,h}=\frac{P\times 0.02289+0.72621}{P\times 0.00396+0.960581}\times \frac{f_{\text{u},\text{p}}}{f_{\text{u},\text{h}}}$$

where *P* is the water-octanol partition ratio and was estimated from the computer-calculated *logP* as neutral (*clogP*):

(A2) $$P=10^{\text{log}\;P}$$

*f_u,h_* is the hepatic unbound fraction for a specific binding on albumin, globulins, and lipoproteins. The tissue interstitial fluid-to-plasma concentration ratios of albumin, globulins, and lipoproteins were assumed to be 0.5:

(A3) $$\frac{f_{\text{u},\text{p}}}{f_{\text{u},\text{h}}}=0.5\times \left(f_{\text{u},\text{p}}+1\right)$$

## Appendix B

The initial parameter values of CL_h,int_′ and V_1_′ used to execute the fitting calculation of the PBPK model with WinNonlin software were derived from the follow equations.

Hepatic clearance (CL_h_) was estimated from Equation B1, which was derived from Equation B2:

(B1) $${CL}_{h}=\frac{\text{Dose}\times Q_{h}-\text{AUC}\times {CL}_{r}\times Q_{h}}{\text{AUC}\times Q_{h}+\text{Dose}}$$

(B2) $$\frac{{CL}_{\text{tot}}}{F}=\frac{{CL}_{n}+{CL}_{r}}{1-\frac{{CL}_{h}}{Q_{h}}}=\frac{\text{Dose}}{\text{AUC}}$$

where *AUC* is the area under the curve.

The bioavailability (F), fraction absorbed (F_a_), and intestinal availability (F_g_) are related as:

(B3) $$F=F_{a}\times F_{g}\times F_{h}$$

where *F_h_* is fraction unmetabolized in the liver.

In this study, we assume *F_a_F_g_* = 1.0; then, the bioavailability was calculated from Equation B4. (The prime represents the value under the assumption of *F_a_F_g_* = 1.0):

(B4) $$F^{\prime }=F_{a}F_{g}\times F_{h}=F_{h}=1-\frac{{CL}_{h}}{Q_{h}}$$

The initial value of V_1_ was estimated from Equation B5 using the fitted calculation results of the one-compartment model (V_d_/F) and the F′ value from Equation B4:

(B5) $$V_{1}^{\prime }=(V_{d}/F)\times F^{\prime }$$

The initial value of hepatic intrinsic clearance (CL_h,int_) was estimated from Equation B6, where CL_h_ was evaluated from Equations B7,8:

(B6) $${CL}_{h,\text{int}}^{\prime }=\frac{R_{b}}{f_{u,p}}\times \frac{Q_{h}\times {CL}_{h}}{Q_{h}-{CL}_{h}}$$

(B7) $${CL}_{\text{tot}}=(V_{d}/F)\times K_{el}\times F^{\prime }$$

(B8) $${CL}_{h}={CL}_{\text{tot}}-{CL}_{r}$$

Values of adjusted distribution volume (V_d_/F) and elimination constant (K_el_) were calculated from the fitting calculation of the one-compartment model, and Equation B7 was derived from Equation B9:

(B9) $$\frac{{CL}_{\text{tot}}}{F}=\left(V_{d}/F\right)\times K_{el}$$

The initial value of k_a_ for the fitting calculation was used as the primary results of WinNonlin with the one-compartmental model.

## Appendix C

The parameter values of CL_r_, k_a_, and V_1_ in the human PBPK model were estimated using a scale-up strategy from rats to humans as follows. Human renal clearance CL_r,human_ was estimated from Equation C1, which was derived from Equation C2, where *BW_rat_* = 0.25 kg and *BW_human_* = 70 kg:

(C1) $${CL}_{r,\text{human}}=\frac{{CL}_{r,\text{rat}}}{{BW}_{\text{rat}}^{2/3}}\times {BW}_{\text{human}}^{\frac{2}{3}}$$

(C2) $${CL}_{r}=a\times {BW}^{\frac{2}{3}}$$

The human systemic circulation volume (V_1,human_) was estimated from Equations C3 and C4, where V_h,human_, blood volume (V_b,rat_), and V_b,human_ were 1.5 L, 0.016 L, and 4.9 L, respectively:

(C3) $$V_{1,\text{human}}=V_{d,\text{human}}-V_{h,\text{human}}\times \frac{K_{p,h}\times F_{h}}{R_{b}}$$

(C4) $$V_{d,\text{human}}=V_{b,\text{human}}+\left(V_{d,\text{rat}}-V_{b,\text{rat}}\right)\times \frac{R_{b,\text{rat}}}{f_{u,p,\text{rat}}}\times \frac{f_{u,p,\text{human}}}{R_{b,\text{human}}}$$

where physicochemical parameters such as K_p,h_, R_b_, and f_u,p_ were assumed to be consistent between rats and humans The derivation of K_p,h_ is shown in Appendix A.

The human absorption rate constant (k_a_) was estimated from Equation C5 [41]:

(C5) $$k_{a,\text{human}}=0.744\times k_{a,\text{rat}}$$
